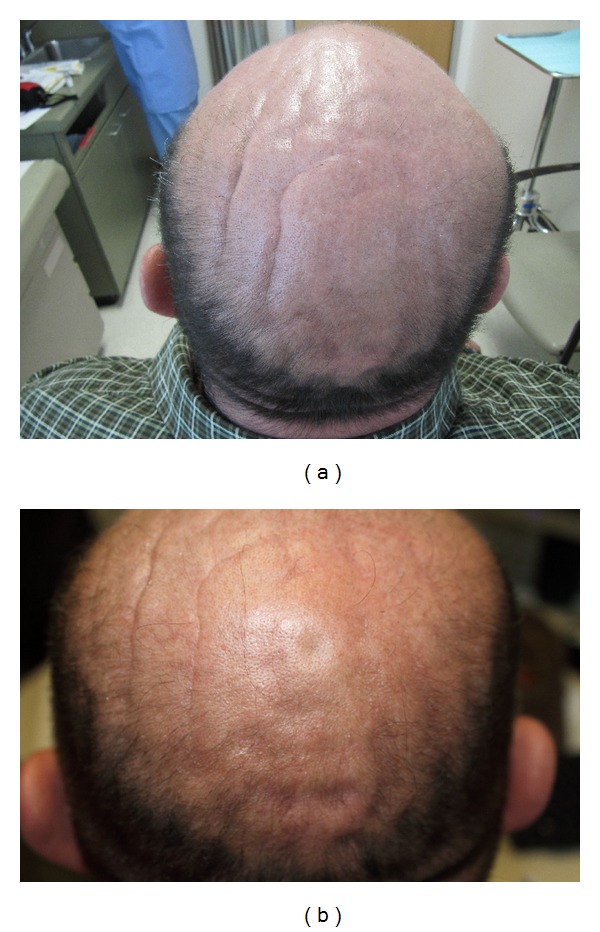# Cutis Verticis Gyrata in Men Affected by HIV-Related Lipodystrophy

**DOI:** 10.1155/2013/941740

**Published:** 2013-09-12

**Authors:** Keshav Khanijow, Patrick Unemori, Kieron S. Leslie, Kathleen Mulligan, Morris Schambelan, Toby Maurer

**Affiliations:** ^1^Department of Dermatology, UCSF School of Medicine, 1701 Divisadero Street, 3rd Floor, San Francisco, CA 94115, USA; ^2^Department of Medicine, Endocrinology, UCSF School of Medicine, SFGH Building 30, 1001 Potrero Avenue, San Francisco, CA 94110, USA

## Abstract

We report the occurrence of cutis verticis gyrata (CVG), a disfiguring dermatological condition, in four patients with HIV-related lipodystrophy (HIVLD). These four patients had abnormal metabolic and hormonal lab values which we compare with metabolic and hormonal perturbations cited in previous HIVLD cohorts. In addition, we describe the sole use of poly-L-lactic acid as a potential treatment for decreasing the appearance of CVG-associated ridges.

## 1. Introduction

HIV-associated lipodystrophy (HIVLD) is a condition characterized by an atypical distribution of body fat and metabolic abnormalities including insulin resistance and dyslipidemia. It is estimated to affect 13–62% [1–3] of HIV-positive patients on highly active antiretroviral therapy (HAART) and occurs more commonly with regimens including thymidine analog nucleoside reverse transcriptase inhibitors (NRTIs) and selected protease inhibitors (PIs) [4].

We report four patients with HIVLD who developed cutis verticis gyrata (CVG). Our clinic sees approximately 1,000 HIV-positive males each year. This is the first case series associating these two conditions. CVG is a dermatological condition defined by the formation of ridges on the scalp with a cerebriform or gyrate appearance [5, 6], Figure 1(a). In its primary form, it predominantly affects males, has a post-pubertal onset, and is associated with various neuropsychiatric conditions [7]. In contrast, secondary CVG manifests asymmetrically, has a variable age of onset, and has been linked with various metabolic and hormonal disturbances such as hypothyroidism [5], diabetes mellitus [7], corticosteroid treatment [6], and anabolic steroid usage [8]. Although secondary CVG has a relatively low prevalence, with less than 500 cases reported prior to 2003 worldwide [6], it is disfiguring and associated with other conditions that could have serious long-term metabolic consequences. Currently, surgical resection is the main treatment for reducing the appearance of CVG-associated scalp folding [9, 10].

## 2. Materials and Methods

Patients were seen in the Dermatology Clinic in San Francisco General Hospital and referred to the hospital's Genera Clinic Research Center. Patients were evaluated with a standardized intake questionnaire, fasting blood samples, and DEXA scans. 

## 3. Results and Discussion

All four patients were male. At the time of presentation, their ages were 50, 52, 59, and 61, and they had been aware of their HIV-positive status for 16, 13, 30, and 16 years, respectively. Their most recent CD4 counts were 470, 531, 399, and 560 cells/mm^3^, and all patients had undetectable viral loads. Previous antiretroviral regimens included NRTIs (*n* = 4), nonnucleoside RTIs (*n* = 2), PIs (*n* = 2), integrase inhibitors (*n* = 3), and a fixed-dose combination of emtricitabine/tenofovir/efavirenz (Atripla, *n* = 1). Medications of particular interest that were used by some patients prior to CVG development included the thymidine NRTIs stavudine (*n* = 2) and zidovudine (*n* = 3), which are commonly associated with the adverse effect of lipodystrophy [11]. In addition, one patient had previously taken testosterone for more than 10 years. Medications of interest that were started after CVG development included testosterone replacement for confirmed hypogonadism (*n* = 2 for ≤2 years) and growth hormone (*n* = 1 for 1 year). All patients denied use of other anabolic steroids. Median body mass index (BMI) was 33.5 kg/m^2^ with all subjects having an elevated BMI (overweight, 25–29, *n* = 1; obese class I, 30–35, *n* = 2; obese class II, 35–39, *n* = 1). Manifestations of lipodystrophy included facial lipoatrophy (*n* = 4) and increased dorsocervical fat deposits (*n* = 2). 

The patients first noticed their scalp ridging at variable times (more than 10 years previously, *n* = 3; two years previously, *n* = 1). In all cases, scalp ridging was noticed after HIVLD development. The patients presented with either all transverse ridges (*n* = 3, median 9, and range 2–12) or all longitudinal ridges (*n* = 1, 4 ridges) not obliterated by traction or extension. All clinical profiles were consistent with secondary CVG, given postpubertal onset and lack of other conditions traditionally associated with primary CVG such as neuropsychiatric disorders. None of the patients had sought prior treatment for reduction of their scalp ridging.

Metabolic profiles were obtained and compared with both standard reference ranges and mean lab values from the lipodystrophy case definition study, which examined 265 cases [12]. Our patients' total protein, bilirubin, albumin, alkaline phosphatase, and cholesterol measurements were within normal limits. However, our patients did have impaired (5.6–7.0 mmol/L; *n* = 3) or diabetic range (>7.0 mg/dL; *n* = 1) fasting glucose levels. In comparing data from our CVG patients to the HIVLD case definition study, our patients had lower total cholesterol levels (4.5 versus 5.6 mmol/L [12]), comparable mean HDL cholesterol levels (1.0 versus 1.1 mmol/L [12]), higher mean fasting glucose levels (6.8 versus 5.3 mmol/L [12]), and higher mean triglyceride levels (4.1 versus 3.4 mg/dL [12]). In both groups, mean triglyceride levels were not within standard reference ranges.

LH, TSH, testosterone, and estradiol levels were all within normal limits. Fasting insulin levels were measured in three patients, and all three had elevated fasting insulin levels (156, 294, and 480 mU/L) compared to both the standard reference range (18–150 mU/L) and the mean value reported in the HIVLD case definition cohort (117 mU/L [12]).

Two of our patients had total and regional fat content measured using dual-energy X-ray absorptiometry (DEXA). The trunk to limb fat ratios of our patients (3.0, 3.7) were higher than the ratios for both HIVLD patients (1.89) [12] and HIV-positive controls (1.16) [12] from another study, implying that our CVG cohort had either greater central fat deposition, greater limb atrophy, or a combination of the two. Our patients' total limb fat (4.7 kg, 6.7 kg) was lower than the HIV uninfected male/s' (7.2 kg) [13] but higher than the HIVLD patients' total limb fat (3.1 kg) [13]. 

As an alternative to surgical management with its operative risks, one patient was treated with poly-L-lactic acid (Sculptra) injected into two scalp furrows. It was hypothesized that the resulting collagenesis from injection of the polymer could be used to fill the depressions created by the CVG-associated ridges. Two furrows on this patient as seen in Figure 1(a) were treated with two courses of poly-L-lactic acid injections, five weeks apart. The patient tolerated the treatment well with no unexpected side effects. A 10-month follow-up visit showed the ridges to be decreased in depth, Figure 1(b).

## 4. Conclusion

In summary, our patients with CVG, who also have HIVLD (clinically diagnosed facial lipoatrophy and objective evidence of obesity and central fat accumulation), appear to have higher fasting glucose, triglyceride, and insulin levels and a higher trunk fat : limb fat ratio than that of HIVLD patients in the HIVLD case definition study [12]. CVG has a relatively low prevalence, with less than 500 cases reported prior to 2003 [7]. As such, it is unusual to observe CVG in any cluster of patients, and it is likely that these patients' HIVLD contributed to their CVG development. Nevertheless, this study was limited by a small sample size, and further prospective studies are needed to more clearly define a possible link between CVG pathogenesis and HIV-related lipodystrophy. Although surgical resection is the current treatment for CVG-associated scalp ridging, poly-L-lactic acid injections can be considered for further study as an alternative treatment.

## Figures and Tables

**Figure 1:**